# Sialic Acid Linkage Analysis Refines the Diagnosis of Ovarian Cancer

**DOI:** 10.3389/fonc.2019.00261

**Published:** 2019-04-11

**Authors:** Tereza Dědová, Elena Iona Braicu, Jalid Sehouli, Véronique Blanchard

**Affiliations:** ^1^Institute of Laboratory Medicine, Clinical Chemistry and Pathobiochemistry, Charité - Universitätsmedizin Berlin, Corporate Member of Freie Universität Berlin, Humboldt-Universität zu Berlin, and Berlin Institute of Health, Berlin, Germany; ^2^Department of Biology, Chemistry and Pharmacy, Freie Universität Berlin, Berlin, Germany; ^3^Department of Gynecology, European Competence Center for Ovarian Cancer, Charité - Universitätsmedizin Berlin, Corporate Member of Freie Universität Berlin, Humboldt-Universität zu Berlin, and Berlin Institute of Health, Berlin, Germany

**Keywords:** *N*-glycans, sialic acids, 2–3 sialic acids, sialic acid linkage, ovarian cancer, biomarker, MALDI-TOF

## Abstract

Epithelial ovarian cancer (EOC) is a rather rare but lethal disease that is usually diagnosed at an advanced stage; this is due to a lack of early diagnostic markers. At the time being, less than a quarter of patients are diagnosed when the tumor has not metastasized yet. In previous work, we demonstrated that antennarity, fucosylation, and sialylation increased in EOC patients and built a glycan-based score that was able to diagnose EOC better than CA125, the routine diagnostic marker, does. To date, little attention had been paid to the sialic acid linkages of *N*-glycans in the context of blood biomarker research. In this work, the sialic acid linkages of the serum glycome of ovarian cancer patients were investigated for the first time by MALDI-TOF-MS. To this end, we released *N*-glycans, derivatized sialic acids solely in a linkage-specific way and measured glycome profiles by MALDI-TOF mass spectrometry. A statistically significant decrease was observed between late stage patients and controls or early stage patients for high-mannose, hybrid-type, complex-type asialylated, bi, tri- and tetraantennary sialylated structures. A significant decrease of monosialylated monoantennary N-glycan structures was observed in early and late stage EOC when compared to healthy controls. Statistically significant increases were observed in early and late stage patients compared to controls for tri, tetraantennary fucosylated structures, afucosylated, and fucosylated triantennary structures taken as α-2,3-linked/α-2,6-linked sialic acid ratio. Moreover, all afucosylated and fucosylated structures taken as α-2,3-linked/α-2,6-linked sialic acid ratio and the α-2,3-linked/α-2,6-linked sialic acid ratio of all sialylated structures were increased significantly for early and late stage EOC patients when compared to healthy controls. Finally, ROC curves were built for the most significant glycan combinations and we were able to show that the serum glycome sialic acid ratio could enhance ovarian cancer diagnosis as sialic acid linkage modulations arise even in early stage ovarian cancer.

## Introduction

Ovarian cancer was among five leading cancer types in cancer deaths in women in United States in 2017 (1) with estimated 14,800 ovarian cancer deaths and 22,440 new cases. The 5-year relative survival rate ranges from 92% in early stage to only 29% in later stages. Currently, only about 20–25% of ovarian cancer patients are diagnosed in early stages (2). Early diagnosis is therefore crucial for the long-term survival rate, however 60% of cases in United States between 2006 and 20012 were of late stage (1). There are three types of cells, from which most of benign and malignant ovarian tumors originate, namely epithelial, stromal and germ, with epithelial being the most common (>90%) (3). The routinely used tumor marker for ovarian cancer CA125 shows specificity of 94–98.5%, but low sensitivity (50–62% for early stages of epithelial ovarian cancer) (4). Since there are no early warning signs, there is a pressing need for improvements in early stage diagnostics (5). Newer biomarkers have been proposed and used, such as HE4, which shows better sensitivity than CA125 in terms of distinguishing benign disease from malignant tumor (6). However, the most promising approach seems to be the use of multi-factor diagnostics, such as a combination of CA125, HE4 and so-called Symptom index (SI) (7), which has a sensitivity of 84% and a specificity of 98.5% (8). Alternative methods for early ovarian cancer diagnosis have been proposed in the last decade, such as microRNA (9), protein panel screening (10, 11) and bioinformatic tools (12).

For the potential improvements in ovarian cancer diagnostics, there is a need to understand causes and pathological alterations of this malignancy. Glycosylation plays an important role in biological processes, such as cell recognition, cell-cell interactions, cell-cell communication and adhesion (13). On human glycoproteins, sialic acids are either α-2,3- or α-2,6-linked to galactoses and are the most exposed monosaccharides to the outer environment, and as such participate in biological processes including cancerogenesis (14). Correlation were made between increased sialylation and ovarian cancer stages (5, 15–17), however, the linkage type has never been investigated in detail.

Biskup et al. recently identified characteristic changes of the serum glycome that were combined in a score named GLYCOV that could diagnose primary epithelial ovarian cancer in a better way than CA125 (5, 17). GLYCOV contains seven sialylated *N*-glycans but the type of sialic acid linkage has not been studied yet. A cohort of 110 patients including early (FIGO stages I + II) and late stages (FIGO III and IV) as well as age-matched controls was enrolled in this work. Glycoproteins from serum were released by PNGase F. Thereafter, the *N*-glycan pool was derivatized using linkage-specific labeling and measured by MALDI-TOF-MS in order to study how the α-2,3/α-2,6 sialic acid ratio evolves with cancer progression.

## Materials and Methods

### Sample Collection

Serum samples from 110 women aged 32–81 years (mean = 57.1, median = 55.5 years) were used in this study (Table 1). There were 77 samples from primary serous ovarian cancer patients of epithelial origin and 33 healthy controls. Healthy controls were women who were free from cancer, liver or kidney insufficiency, inflammatory diseases or pregnancy. Blood was collected as a part of the Tumor Bank Ovarian Cancer project (http://www.toc-network.de/), where information about the FIGO stage was obtained. The Charité Medical University approved the use of the samples (EA4/073/06 and EA1/285/09). Clot activator serum tubes (Vacutainer, BD, Medical-Pharmaceutical System, NJ, USA) were used for collection. Blood was allowed to clot for a minimum of 30 min up to 2 h at room temperature and serum was separated by centrifugation at 1,200 g for 15 min. The CA125 II immunoassay was used to measure CA125 on a COBAS 6000 analyser (Roche Diagnostics, Germany). Serum was aliquoted and stored at −80°C until the time of glycan analysis.

**Table 1:** Demographics of samples used in this study. CA125 are shown as kU/L.

**Stage**	**Control** ***N*** **=** **33**		**Early stage**				**Late stage**			
			**FIGO stage I** ***N*** **=** **10**		**FIGO stage II** ***N*** **=** **9**		**FIGO Stage III** ***N*** **=** **43**		**FIGO Stage IV** ***N*** **=** **15**	
**Demographics**	**Age**	**CA125**	**Age**	**CA125**	**Age**	**CA125**	**Age**	**CA125**	**Age**	**CA125**
Mean	52.0	14.5	57.6	514.9	57.4	300.6	59.9	1503.6	59.9	2065.3
Median	50.0	12.0	59.5	28.5	53.0	190.0	60.0	616.0	58.0	1195.0
Range	40–81	6–38	48–67	6–4841	40–78	23–851	35–79	11–8094	32–81	6–14310

### N-Glycan Release

Two microliters of human serum were denatured for 10 min at 50°C in the presence of four microliters of 1% SDS (w/w) (Merck Millipore, Germany). Thereafter, four microliters of a releasing solution containing 2% NP-40 (Calbiochem, CA, USA) (w/w) and 0.5 mU PNGase F (EC 3.5.152; Roche Applied Science, Indianapolis, IN) in 2.5 × PBS (10xPBS containing 57 g/L Na_2_HPO_4_ × 2H_2_O, 5 g/L KH_2_PO_4_, and 85 g/L NaCl, pH 7.4) were added to the samples and incubated for 5 h at 37°C. Samples were then stored at −20°C until sialic acids were labeled.

### Sialic Acid Derivatization

An (α2-3) and (α2-6)-linkage specific sialic acid derivatization was performed. To one microliter of *N*-glycan release digest were added 10 μl of reaction mixture consisting of 250 mM dimethylamine, 500 mM 1-hydroxybenzotriazole hydrate, 250 mM 1-ethyl-3-(3-(dimethylamino)propyl) carbodiimide in DMSO) and incubated for 2.5 h at 60°C. This step resulted in the dimethylamidation of the carboxyl groups of 2,6-linked sialic acids whereas the carboxyl groups of 2,3-linked sialic acids reacted with the adjacent galactoses to form an unstable lactone. Then, four microliters of 28% ammonium hydroxide were added and samples were incubated for additional 2.5 h at 60°C, which resulted in the hydrolysis of lactones into amides (18). Samples were then adjusted to 92% ACN and transferred to −20°C for 15 min prior to purification.

### Purification (19)

First, each well of an AcroPrep^TM^ Advance 96-well filter plate containing a 0.45 μm GHP filter membrane was washed with 200 μl of 70% cold ethanol, followed by 3 × 200 μl MilliQ water and 3 × 200 μl of 96% ACN. Samples were then applied and incubated for 10 min, after which a low vacuum was applied to ensure a slow flow through the membrane. Each well was then washed with 3 × 200 μl of cold 96% ACN. Elution of *N*-glycans from the plate was performed by addition of 2 × 50 μl of MilliQ water. Samples were then dried in a vacuum centrifuge and stored until MALDI-TOF-MS measurement.

### MALDI-TOF-MS and Data Analysis

MALDI-TOF-MS spectra were recorded on an Ultraflex III mass spectrometer (Bruker Daltonics, Bremen, Germany) equipped with Smartbeam laser (100 Hz laser frequency) in reflectron positive mode as a sum of 2500 laser shots in the mass range 1,200–5,000 Da using 25 kV accelerating voltage and ion suppression bellow 1,190 Da. Raw spectra were exported as ASCII text files and Massy Tools script was first used to re-calibrate the obtained spectra using list of 10 glycan masses (N4H3F1, N4H4F1, N4H5D1, N4H5D1F1, N4H5D1A1, N4H5D2, N4H5D2F1, N5H6D2A1, N5H6D2A1F1, N6H7D2A2).

The program mMass was then used to sum all spectra from each sample (*n* = 4) leading to a sensitivity increase especially in the high-mass region. The Massy Tools Python script (20) was then used on these summed spectra for a targeted peak extraction of background subtracted analyte areas of 100 glycan structures. The output of targeted peak extraction was then processed further, by removing all structures, which had signal-to-noise ratio (S/N) <9 for more than 90% of all collected spectra. Additionally, all these structures were evaluated for separate sample groups and only structures, which appeared in more than one third of samples in at least a single sample group, were used for statistical analysis, resulting in areas under the curve for 72 N-glycan peaks. The extracted values were then normalized so that the intensity of the peak at *m/z* 2299.9 (H4H5D2) was set to 100%. As a result of this approach, the variance of this structure could not be statistically tested.

### Glycan Traits and Statistical Analysis

Total intensities of specific glycan traits, namely high-mannosylation, fucosylation, linkage-specific sialylation and antennarity, were calculated as a sum of intensities of all respective structures (Supplementary Table 1). For the calculation of sialylation traits, firstly, relative sialylation intensities were calculated for each structure as shown in Equation 1:

**S (degree of sialylation)** **=** D_n_ + A_n_
**relative** **α-2,6 sialylation** **=** (D_n_/S) ^*^ (relative intensity N_n_H_n_D_n_A_n_F_n_)
**relative** **α-2,3 sialylation** **=** (A_n_/S) ^*^ (relative intensity N_n_H_n_D_n_A_n_F_n_)

where H = hexose, N = N-acetylhexosamine, A = α-2,3-linked sialic acid, D = α-2,6-linked sialic acid, F = fucose

Then, total sialylation traits were calculated as a sum of relative sialylation intensities of the respective structures. For example, the total α-2,3-sialylation of triantennary fucosylated *N*-glycans was calculated according to Equation 2:

**Fucosylated complex triantennary** **α-2,3** = (1/2)^*^(N5H5D1A1F1 + N5H6D1A1F1) + (2/3)^*^(N5H6D1A2F1) + (1/3)^*^(N5H6D2A1F1)

where H = hexose, N = N-acetylhexosamine, A = α-2,3-linked sialic acid, D = α-2,6-linked sialic acid, F = fucose

Additionally, a ratio between α-2,3 and α-2,6 sialylation was calculated for each grouped *N*-glycans, which included all complex sialylated structures (Supplementary Table 1).

Means and standard deviations were calculated for FIGO stages and for grouped ovarian cancer stages (healthy controls, early stage = FIGO I + FIGO II, late stage = FIGO III + FIGO IV). The Shapiro-Wilk test showed that the data were not normally distributed and therefore non-parametric tests were used for further statistical evaluation. The Jonckheere-Terpstra test (T_JT_) was selected since the independent variables consisted of three ordinal groups of cancer progression (healthy control < early stage < late stage), while the dependent variables were measured on a continuous level, and there was no relationship between samples in various groups. The T_JT_ was used to test the null hypothesis that the distribution of individual *N*-glycans was the same across ovarian cancer stages. In other words, it was tested if there is a positive or negative trend during cancer progression. The null hypothesis was rejected when *p* < 0.05.

## Results

In this work, the sialic acid linkages of the serum glycome of ovarian cancer patients were investigated for the first time by MALDI-TOF-MS. To this end, serum *N*-glycans from ovarian cancer patients and age-matched healthy controls were released after serum protein denaturation. A linkage-specific sialic acid derivatization was performed, whereby the carboxyl groups of 2,6-linked sialic acids were dimethylamidated and the carboxyl groups of 2,3-linked sialic acids were first lactonized then amidated (18). Samples were finally purified using HILIC 96-well plates, dried by vacuum concentration and measured by MALDI-TOF-MS.

### N-glycan Features of the Total Serum Glycome

**Figure 1:**
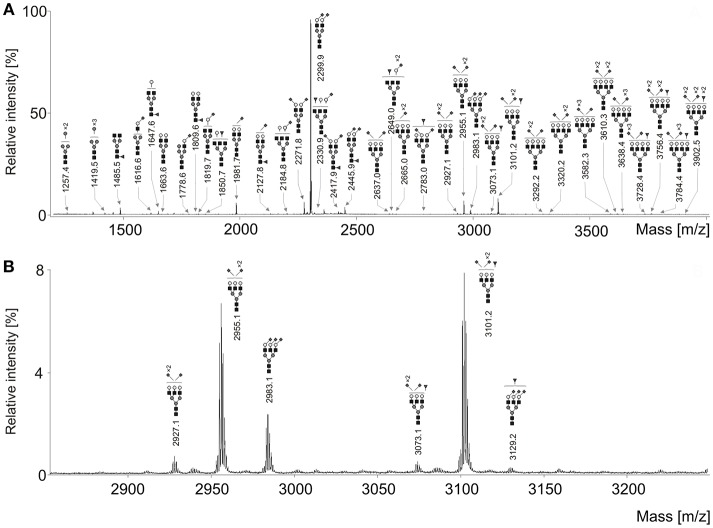
**(A)** Representative MALDI-TOF mass spectrum of the serum N-glycans of an ovarian cancer patient after linkage-specific sialic acid derivatization. **(B)** Enlargement of the region 2,850–3,250 Da. Measurements were performed in the positive-ion mode and all molecular ions are present in sodiated form [M + Na]^+^. Dark gray circle, Man; light gray circle, Gal; black square, GlcNAc; dark gray triangle, Fuc; dark gray diamond, Neu5Ac.

Seventy-one *N*-glycan structures were detected (Figure 1), four were high-mannoses, thirteen were asialylated complex-type structures, of which seven were fucosylated. Due to sialic acid linkage-specific labeling, overall 54 different sialylated *N*-glycans were detected, of which 24 were fucosylated. It should be noted that many of these structures were not observed in early stages of ovarian cancer and/or healthy controls. However, all these structures were included in statistical analysis due to their possible biological significance.

Relative intensities of 71 glycans were normalized to the base peak intensity. Means and standard deviations (SD) were calculated for healthy controls and all FIGO stages and are presented in Table 2. Thereafter, *N*-glycans were grouped according to type, antennarity, fucosylation and sialylation. Moreover, their relative abundances were statistically compared between healthy controls, early and late stages, whereby the *post-hoc* statistical test is reported for the total traits only (Supplementary Tables 2–10).

**Table 2:** Mean relative intensities (normalized to the base peak intensity) and SD of all detected structures across ovarian cancer stages measured by MALDI-TOF-MS.

	**Glycan**	**Mass *[m/z]***	**Healthy control**		**Figo I**		**Figo II**		**Figo III**		**Figo IV**	
			**Mean**	**SD**	**Mean**	**SD**	**Mean**	**SD**	**Mean**	**SD**	**Mean**	**SD**
High-mannose	N2H5	1257.4	2.06	1.31	2.48	0.69	1.39	0.48	0.86	0.45	0.75	0.44
	N2H6	1419.5	1.46	1.02	1.99	0.72	1.36	0.46	0.95	0.51	0.87	0.58
	N2H7	1581.5	0.19	0.19	0.50	0.25	0.32	0.15	0.25	0.20	0.24	0.20
	N2H8	1743.6	0.37	0.21	0.61	0.37	0.49	0.17	0.40	0.24	0.36	0.24
Complex	N3H4	1298.4	0.18	0.21	0.25	0.24	0.15	0.16	0.07	0.10	0.08	0.09
	N4H3	1339.5	0.37	0.70	0.53	0.34	0.29	0.26	0.23	0.26	0.23	0.24
	N4H5	1663.6	0.73	0.43	0.53	0.12	0.38	0.18	0.24	0.16	0.23	0.15
	N5H4	1704.6	0.16	0.29	0.36	0.18	0.20	0.10	0.23	0.18	0.21	0.16
	N5H5	1866.7	0.04	0.07	0.14	0.17	0.08	0.08	0.09	0.11	0.07	0.09
	N5H6	2028.7	0.00	0.01	0.02	0.06	0.01	0.04	0.02	0.04	0.03	0.05
Complex fucosylated	N4H3F1	1485.5	5.22	3.15	10.49	4.51	8.05	6.05	9.19	12.36	6.20	4.05
	N4H4F1	1647.6	5.19	2.51	8.16	3.93	4.99	3.13	4.45	3.89	3.58	2.37
	N4H5F1	1809.6	1.60	0.89	1.98	1.33	1.23	1.05	0.90	0.84	0.72	0.62
	N5H4F1	1850.7	1.03	0.43	1.42	0.54	0.86	0.40	0.77	0.61	0.63	0.42
	N5H5F1	2012.7	0.15	0.10	0.29	0.20	0.13	0.11	0.14	0.15	0.13	0.10
	N5H5F2	2158.8	0.50	0.13	0.72	0.21	0.54	0.17	0.56	0.26	0.59	0.24
	N5H6F1	2174.8	0.01	0.02	0.07	0.12	–	–	0.03	0.05	0.01	0.02
Complex sialylated	N3H4A1	1588.6	0.01	0.02	0.54	0.79	0.21	0.25	0.46	0.47	0.42	0.48
	N3H4D1	1616.6	1.75	0.51	1.33	0.33	0.89	0.21	0.82	0.33	0.88	0.33
	N3H5A1	1750.6	1.93	0.75	2.03	0.60	1.63	0.46	1.11	0.54	0.85	0.61
	N3H5D1	1778.6	0.47	0.20	0.49	0.08	0.33	0.08	0.26	0.13	0.25	0.10
	N4H4A1	1791.6	0.04	0.16	0.77	1.10	0.32	0.36	0.68	0.70	0.58	0.68
	N4H4D1	1819.7	0.71	0.28	0.73	0.30	0.46	0.12	0.53	0.28	0.52	0.28
	N3H6A1	1912.7	0.02	0.08	0.11	0.15	0.01	0.04	0.05	0.09	0.05	0.10
	N3H6D1	1940.7	0.10	0.08	0.12	0.07	0.05	0.06	0.09	0.07	0.07	0.06
	N4H5A1	1953.7	0.28	0.13	0.54	0.44	0.28	0.16	0.43	0.32	0.37	0.32
	N4H5D1	1981.7	12.00	2.65	10.52	2.12	8.38	2.60	7.51	2.89	8.32	3.13
	N5H5D1	2184.8	0.36	0.22	0.51	0.20	0.31	0.15	0.25	0.15	0.27	0.13
	N4H5A2	2243.8	0.09	0.10	0.14	0.11	0.04	0.08	0.02	0.05	0.04	0.05
	N4H5D1A1	2271.8	3.77	1.27	3.80	0.64	3.26	1.14	3.09	1.08	2.99	1.02
	N4H5D2	2299.9	100.00	–	100.00	–	100.00	–	100.00	–	100.00	–
	N5H6A1	2318.8	0.16	0.29	–	–	0.12	0.14	0.13	0.22	0.11	0.11
Complex sialylated	N5H6D1	2346.9	0.45	0.21	0.44	0.11	0.42	0.15	0.29	0.17	0.32	0.13
	N5H5D2	2502.9	0.27	0.15	0.42	0.11	0.32	0.42	0.21	0.23	0.28	0.28
	N5H6D1A1	2637.0	0.47	0.23	0.49	0.12	0.42	0.22	0.35	0.16	0.32	0.15
	N5H6D2	2665.0	0.64	0.24	0.64	0.16	0.52	0.23	0.39	0.17	0.42	0.11
	N5H6A3	2899.0	–	–	0.04	0.07	–	–	0.03	0.05	0.03	0.05
	N5H6D1A2	2927.1	0.30	0.25	0.32	0.15	0.31	0.21	0.29	0.17	0.26	0.19
	N5H6D2A1	2955.1	6.41	3.07	5.58	2.32	5.22	2.99	4.42	2.35	3.71	2.44
	N5H6D3	2983.1	2.15	0.64	2.93	1.16	2.94	1.65	2.20	0.75	2.28	0.63
	N6H7D1A1	3002.1	0.08	0.10	0.24	0.09	0.22	0.17	0.12	0.09	0.11	0.07
	N6H7D2	3030.1	0.01	0.03	0.07	0.10	0.04	0.08	0.04	0.05	0.05	0.05
	N6H7D1A2	3292.2	0.04	0.06	0.09	0.09	0.09	0.11	0.08	0.09	0.07	0.08
	N6H7D2A1	3320.2	0.05	0.08	0.14	0.12	0.09	0.14	0.09	0.09	0.07	0.06
	N6H7D1A3	3582.3	0.03	0.06	0.05	0.09	0.10	0.14	0.12	0.14	0.11	0.15
	N6H7D2A2	3610.3	0.14	0.12	0.18	0.16	0.24	0.23	0.22	0.17	0.19	0.20
	N6H7D3A1	3638.4	0.01	0.04	0.07	0.09	0.08	0.11	0.08	0.09	0.08	0.08
Complex sialylated fucosylated	N4H5A1F1	2099.7	0.24	0.10	0.23	0.11	0.14	0.12	0.14	0.10	0.17	0.09
	N4H5D1F1	2127.8	2.22	0.84	2.28	1.14	1.55	0.88	1.21	0.75	1.24	0.61
	N5H5D1F1	2330.9	0.94	0.50	1.51	0.89	0.86	0.64	0.67	0.56	0.66	0.31
	N4H5A2F1	2389.9	0.54	0.28	0.62	0.18	0.60	0.20	0.37	0.20	0.22	0.16
	N4H5D1A1F1	2417.9	0.54	0.21	0.66	0.16	0.82	0.55	0.74	0.32	0.91	0.55
	N4H5D2F1	2445.9	4.46	1.15	3.40	1.19	3.56	0.94	2.71	1.05	3.10	1.21
	N5H6D1F1	2492.9	0.05	0.09	0.15	0.15	0.05	0.09	0.02	0.05	0.03	0.05
	N5H5D1A1F1	2621.0	0.01	0.03	0.07	0.10	0.02	0.05	0.03	0.05	0.03	0.04
	N5H5D2F1	2649.0	1.48	0.72	1.55	0.57	1.31	0.80	0.99	0.51	0.93	0.35
	N5H6D1A1F1	2783.0	0.06	0.10	0.18	0.12	0.20	0.14	0.12	0.13	0.19	0.12
	N5H6D2F1	2811.1	0.09	0.10	0.17	0.11	0.17	0.11	0.07	0.09	0.13	0.09
	N5H6D1A2F1	3073.1	0.05	0.07	0.21	0.15	0.25	0.17	0.21	0.13	0.21	0.15
	N5H6D2A1F1	3101.2	2.12	1.06	4.59	2.06	6.37	3.45	5.05	2.46	5.48	2.72
	N5H6D3F1	3129.2	0.12	0.12	0.22	0.14	0.27	0.17	0.15	0.12	0.18	0.11
	N6H7D1A1F1	3148.2	–	–	0.05	0.10	0.06	0.11	0.04	0.07	0.08	0.07
	N5H6D1A2F2	3219.2	–	–	0.01	0.04	0.08	0.12	0.05	0.08	0.08	0.08
	N5H6D2A1F2	3247.2	0.01	0.04	0.04	0.10	0.14	0.14	0.06	0.10	0.11	0.09
	N6H7D1A2F1	3438.3	–	–	–	–	0.07	0.11	0.03	0.06	0.08	0.07
	N6H7D2A1F1	3466.3	–	–	0.01	0.03	0.05	0.11	0.03	0.06	0.06	0.07
	N6H7D1A3F1	3728.4	–	–	–	–	0.11	0.13	0.08	0.12	0.11	0.14
	N6H7D2A2F1	3756.4	0.02	0.04	0.08	0.11	0.25	0.20	0.23	0.19	0.22	0.21
	N6H7D3A1F1	3784.4	–	–	0.01	0.03	0.03	0.06	0.06	0.10	0.08	0.08
	N6H7D1A3F2	3874.4	–	–	0.01	0.04	0.03	0.07	0.03	0.06	0.05	0.08
	N6H7D2A2F2	3902.5	–	–	–	–	0.10	0.18	0.07	0.13	0.11	0.11

The statistical analysis of sialylated complex-type structures was first performed on a single glycan level followed by calculation of glycan traits as explained in the Materials and Methods section. The complex-type sialylated structures are presented here separately based on their fucose content. Additionally, for both fucosylated and afucosylated structures, total glycosylation traits were calculated, such as relative antennarity. It should be noted that while total α-2,6-linked sialylation is pretty similar to a total sialylation relative intensity, the relative α-2,3-linked sialylation shows different patterns (data not shown). Therefore, a ratio between α-2,3-linked sialylation and α-2,6-linked sialylation was calculated. In the following, “total sialylation” refers to the whole sialylation irrespective of the linkage types, whereas “sialylation ratio” refers to the ratio α-2,3-linked /α-2,6-linked sialylated structures as defined in Supplementary Table 1.

### Decrease of N-glycans: High-Mannose, Hybrid, Complex-Type Asialylated, Mono-, Di-, Triantennary Sialylated Structures

#### High-Mannose *N*-glycans

**Figure 2:**
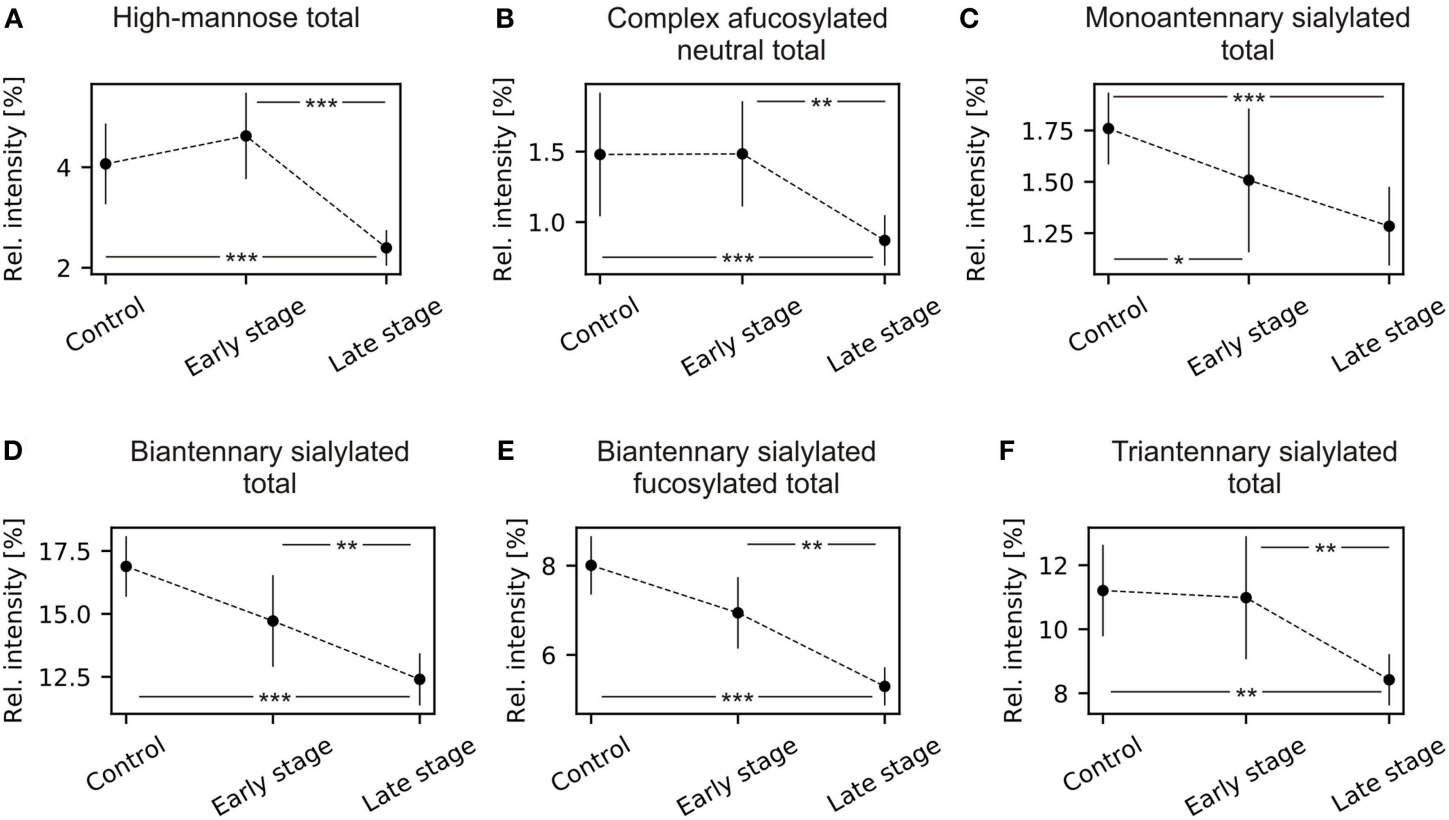
Mean and 95% confidence intervals of *N*-glycans grouped by type, antennarity, sialylation, and fucosylation during ovarian cancer progression. **(A)** High-mannose type total; **(B)** complex type afucosylated neutral total; **(C)** monoantennary sialylated total; **(D)** biantennary complex total; **(E)** biantennary sialylated fucosylated total; **(F)** triantennary sialylated total. ^*^p < 0.05, ^**^p < 0.01, ^***^p < 0.001.

The T_JT_ showed that there was a statistically significant negative trend in mean rank distribution of N2H5 and N2H6 for both grouped (early and late) FIGO stages (Supplementary Table 2). The *post-hoc* analysis of the T_JT_ test revealed that there was a highly significant decrease in total high-mannosylation between healthy controls and late stage patients, and between early stage and late stage patients (Figure 2A). There was no statistically significant difference between healthy controls and early stage patients.

#### Hybrid-Type Sialylated *N*-glycans

Statistical analysis revealed a significant decrease in both α-2,3- and α-2,6-sialylation forms of hybrid structure N3H5S1 (Supplementary Table 3) as well as a significant increase in the α-2,3-isomer of N3H6S1. For the total relative intensity of hybrid structures, there was a statistically significant decrease. The *post-hoc* analysis revealed that there were statistically significant decreases between healthy controls and late stage patients, and between early stage and late stage patients but no statistically significant difference between healthy controls and early stage patients.

#### Neutral Afucosylated Complex-Type *N*-glycans

There were statistically significant differences for all complex-type neutral *N*-glycans, except for N4H3. Similarly to the high-mannosylation, there was a highly significant decrease in total relative intensity of afucosylated complex-type *N*-glycans (Supplementary Table 4) but no statistically significant difference between healthy controls and early stage patients (Figure 2B). Additionally, there was a statistically significant decrease of monoantennary and biantennary *N*-glycans (N3H4, N4H5) and increase of triantennary *N*-glycans (N5H4, N5H5, N5H6).

#### Neutral Fucosylated Complex-Type *N*-Glycans

The complex-type core-fucosylated structures that are present for instance on IgG, namely N4H4F1, N4H5F1 showed statistically significant decreases in relative intensities between healthy controls and ovarian cancer stages, but there was no statistically significant difference for the agalactosylated structure N4H3F1 (Supplementary Table 5).

### Complex-Type Sialylated N-glycans: Monoantennary, Biantennary, Biantennary Fucosylated, and Triantennary

A statistically significant negative trend was observed for N3H4D1, the α-2,6-linked isomer, and a statistically positive trend for N3H4A1, the α-2,3-linked isomer. The total relative intensity for monoantennary sialylated structures decreased in advanced cancer stages (Supplementary Table 6). The *post-hoc* analysis revealed that there were statistically significant decreases between healthy controls and early stage patients, and between healthy controls and late stage patients. There was no statistically significant difference between early and late stage patients (Figure 2C). The total relative intensity for both fucosylated and afucosylated biantennary sialylated glycans and triantennary sialylated significantly decreased in ovarian cancer compared to healthy controls (Supplementary Table 7; Figures 2D–F). Significant differences were observed between healthy controls and late stage patients, between early stage and late stage patients but not between healthy controls and early stage patients.

### Increase of α-2,3/α-2,6-sialylation Ratios, a Marker for Early and Late Stage EOC

#### α-2,3/α-2,6-sialylation Ratios of Biantennary Sialylated N-glycans

**Figure 3:**
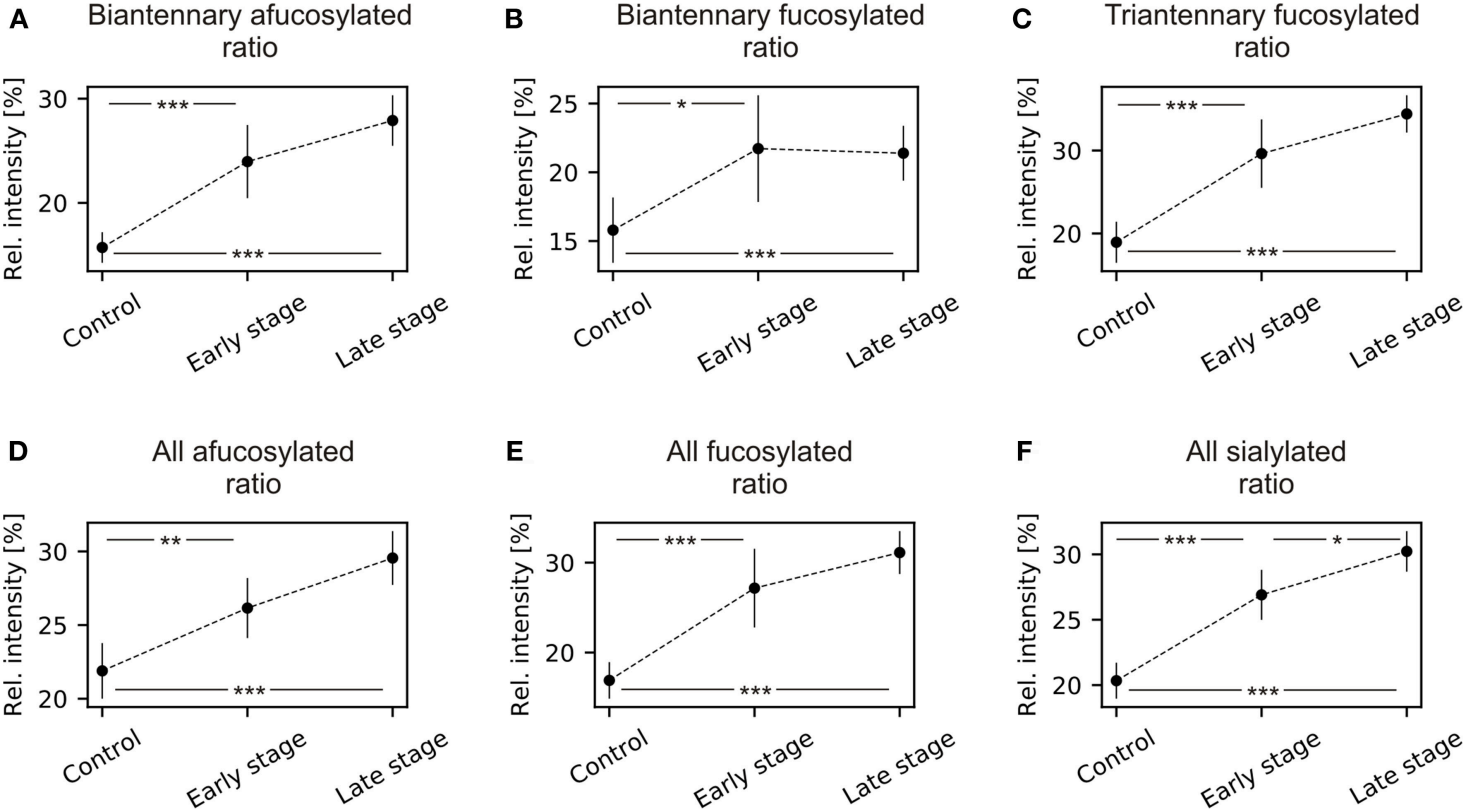
Mean and 95% confidence intervals of α-2,3/α-2,6 ratio of *N*-glycans grouped by antennarity and fucosylation during ovarian cancer progression. **(A)** Biantennary afucosylated ratio; **(B)** biantennary fucosylated ratio; **(C)** triantennary fucosylated ratio; **(D)** all afucosylated ratio; **(E)** all fucosylated ratio; **(F)** all sialylated ratio. ^*^p < 0.05, ^**^p < 0.01, ^***^p < 0.001.

The α-2,3/α-2,6-sialylation ratio significantly increases for both afucosylated and fucosylated *N*-glycans in ovarian cancer patients (Supplementary Table 7; Figures 3A,B). *Post-hoc* analysis showed statistically significant increases between healthy control and early stage and between healthy controls and late stage patients.

#### α-2,3/α-2,6-sialylation Ratios of Triantennary Sialylated N-glycans

There was a statistically significant increase in α-2,3-linked/ α-2,6-linked sialylation ratio of fucosylated triantennary *N*-glycans, which was observed between healthy controls and both early stage and late stage ovarian cancer patients (Figure 3C, Supplementary Table 8).

#### α-2,3/α-2,6-sialylation Ratios of Tetrantennary Sialylated N-glycans

There was a statistically significant increase in relative intensities of three tetraantennary N-glycans, namely N6H7D2, N6H7D1A3 and N6H7D3A1 (Supplementary Table 9) but the ratio between α-2,3 and α-2,6 sialylation did not vary significantly for both afucosylated and fucosylated N-glycans in all three cohorts. This could be due to steric the hindrance of α-2,3-linkages, which theoretically favors mixed α-2,3 / α-2,6 sialic acid linkages.

#### α-2,3/α-2,6-sialylation Ratios of Fucosylated and Afucosylated Stuctures

There were highly significant increases in sialylation ratios for both afucosylated and fucosylated N-glycans, namely between healthy control and both early stage and late stage (Supplementary Table 10; Figures 3D,E). These changes in α-2,3 / α-2,6 sialylation ratio appear to be cancer specific, since in both fucosylated and afucosylated glycans are observed statistically significant differences between healthy controls and cancer stages. Moreover, there were no statistically significant differences between early and late stage ovarian cancer patients, which makes these ratios a possible candidate for improving ovarian cancer diagnostics.

#### α-2,3/α-2,6-sialylation Ratios of All Sialylated N-glycan Structures

On the total sialylation level, when the ratio α-2,3/α-2,6 was used, there were statistically significant increases between all three cohorts, namely between healthy controls and both early and late stages of ovarian cancer and between early and late stage patients (T_JT_ = 743, *z* = 2.269, *p* = 0.035) (Supplementary Table 10; Figure 2F).

#### α-2,3/α-2,6-sialylation Ratios as Enhancement of Ovarian Cancer Diagnosis

Since the changes in sialylation ratio showed statistically significant differences between healthy controls and all primary serous ovarian cancer patients of epithelial origin, the values were used for the construction of receiver operating characteristic (ROC) curves (Supplementary Figure 2), using SPSS for Windows, version 21 (SPSS Inc., Chicago, Ill), to evaluate possible application in ovarian cancer diagnostics. Supplementary Figure 2A shows ROC curves for early stage patients vs. controls whereas Figure 2B presents ROC curves for all EOC patients vs. healthy controls.

The ratios for separate groups based on antennarity and fucosylation showed ROC curves with areas under the curve (AUC) from 0.717 to 0.887, which is a “good” result. The AUC for total sialylation ratio showed the best results for both early stage and all patients: 0.88 (Supplementary Figure 2A) and 0.911 (Supplementary Figure 2B), respectively, however, AUC values were still lower than for the routinely used CA125 (AUC = 0.953). The software MedCalc (Version 18.6) was then used to evaluate the cut-off value of total sialylation ratio based on Youden index (J = 0.7576) and the calculated cut-off value of 0.2424 was estimated (Supplementary Figure 1).

**Figure 4:**
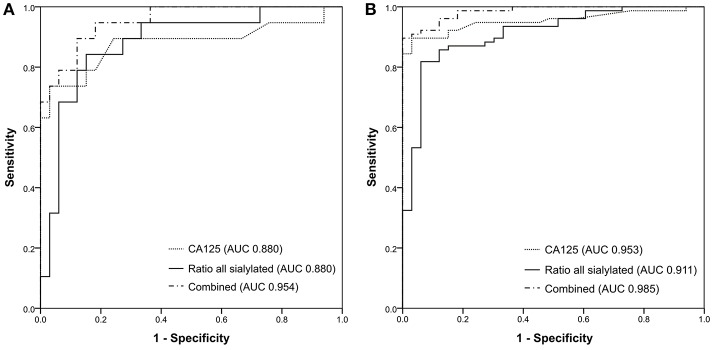
ROC curves generated for **(A)** 33 healthy controls and 19 early-stage patients; **(B)** 33 healthy controls and 77 ovarian cancer patients.

Since the total sialylation ratio showed the best results, it was tested whether the combination of sialylation ratio with CA125 could improve ovarian cancer diagnostic. A logistic regression was performed to evaluate the potential of CA125 and sialylation ratio on the ovarian cancer diagnosis. The prediction model was as follows: −10.553 + 0.108^*^CA125 + 33.062^*^ratio. The logistic regression model was statistically significant [$\chi_{\left(4\right)}^{2}$ = 101.402, *p* < 0.001], explained 85.4% (Nagelkerke *R*^2^) of the variance in ovarian cancer and correctly classified 88.2% of cases. Therefore, ROC curves were plotted for the combined probability results and the final AUC were 0.954 for early stage (Figure 4A) and 0.985 (Figure 4B) for all patients, respectively, which means a “very good” classification.

These results suggest that information about sialylation linkages provides cancer-specific insight and may refine the measurement of CA125 both in early stage and in late stage EOC patients as the combination of both resulted in an improvement of the specificity and the sensitivity.

## Discussion

In the present study, we focused on the analysis of sialic acid linkages from primary serous ovarian cancer patients in early (FIGO I + II) and late stages (FIGO III + IV) of the disease to age-matched healthy controls. *N*-Glycans from serum samples were released by PNGase F and sialic acids were stabilized by linkage-specific labeling (18) before MALDI-TOF-MS measurement.

High-mannose and neutral complex-type N-glycan structures showed statistically significant differences only between healthy patients and late stage ovarian cancer patients and no differences between healthy controls and early stage patients. Additionally, increases of antennarity were observed here that correspond with the published literature, since increased expression of GlcNAcT IV and V leads to increased branching and thus antennarity (21, 22).

Interestingly, in this study, N4H3F1, the agalactosylated N-glycan present on IgG (23), the most abundant serum glycoprotein, did not show any statistical difference whereas its galactosylated forms N4H4F1 and N4H5F1 were significantly decreased. Saldova and colleagues (15) showed using ovarian cancer samples, irrespective of FIGO stages, that there was a decrease of IgG galactosylation and an increase of agalactosyl IgG *N*-glycans in ovarian cancer patients. However, it should be noted that our analysis was performed on 110 whole serum N-glycome samples and not at the IgG level alone. This is the reason why the observed changes can only be partly explained by the IgG glycosylation alone. Compared to the above-mentioned publication, our findings suggest an initial increase of relative abundance of total complex fucosylated *N*-glycans in early FIGO stages, followed by a decrease in late stages. This could be explained by inflammation and greater IgG production in early stages, thus greater abundance of glycan structures carried on IgG.

The majority of sialylation traits showed specificity for ovarian cancer already in early stage, which is desired for potential biomarker use. On the level of total sialylation, regardless of the linkage type, a statistically significant decrease was observed for monoantennary afucosylated *N*-glycans. In contrast, triantennary fucosylated *N*-glycans and tetraantennary *N*-glycans both fucosylated and afucosylated showed statistically significant increase. Since the changes in relative intensities without the focus on sialic acid linkages has been extensively studied in recent years in various malignancies (5, 15, 17, 24–28), these will not be discussed further here.

More importantly, the information about sialic acid linkage ratios always showed statistically significant differences already for early stage EOC. When classified according to the antennarity, there was statistically significant increase in α-2,3/α-2,6 ratio for biantennary afucosylated and fucosylated *N*-glycans, and triantennary fucosylated *N*-glycans. Similarly, there was a statistically significant increase in α-2,3/ α-2,6 ratio of all afucosylated and fucosylated sialylated *N*-glycans. Most importantly, the increase in α-2,3/ α-2,6 ratio of all sialylated *N*-glycans showed statistically significant differences between all three cohorts.

In many publications (5, 17, 25, 27), the glycan structure N5H6S3F1 was significantly increased in malignancies. This structure has four potential sialylation isomers, namely N5H6A3F1, N5H6A2D1F1, N5H6A1D2F1, and N5H6D3F1. Interestingly, in the present study only structures with mixed sialylation, e.g., N5H6A2D1F1, N5H6A1D2F1 show statistically significant increase in ovarian cancer. On the other hand, the structure N5H6D3F1 showed no differences between healthy controls and ovarian cancer patients and N5H6A3F1 could not be detected in any cohort. The increase of N5H6A1D2F1 is accompanied by statistically significant decrease of N5H6A1D2. These changes correlate with findings that synthesis of SLe^x^ antigen requires first addition of sialic acid, followed by addition of antennary fucose (29).

The observed changes in ovarian cancer serum sialylation agree with findings in other types of cancer. Holst et al. showed by MALDI-Imaging in colorectal carcinoma, that the α-2,3-linked sialic acid was increased in stroma, tumor, and necrotic cell regions, while α-2,6-linked sialic acid was more prominent in inflammatory areas, e.g., rich in collagen, necrotic regions and red-blood cells (18). On some colorectal cancer cell lines (HT29, WiDr, SW48, T84, and Lovo) an increased α-2,3-sialylation was observed together with multiple fucosylation (30). Saldova et al. observed increased α-2,3-sialylation in prostate cancer as compared to benign hyperplasia (31). In the study performed by Wang and colleagues (32), increased mRNA expression of ST3Gal III, ST3Gal IV, and ST3Gal VI was observed in ovarian serous carcinoma tissues. Moreover, immunohistochemical staining using the lectin *maackia amurensis agglutinin* showed strong positivity in ovarian epithelial carcinoma part, while normal epithelial part was not. Wen et al. then studied expression of ST3Gal I in serous type epithelial ovarian cancer (33) and in clear cell type epithelial ovarian cancer (34). They proposed α-2,3-sialylation as a potential prognostic marker and a possible therapy target of ovarian cancer.

The sialylation changes were reported here as a ratio between relative α-2,3-sialylation and α-2,6-sialylation. In general, glycans containing exclusively α-2,3-linked sialic acids were not observed in the samples in high amounts and structures carrying both linkage types were most prominent in tri- and tetraantennary structures. This is most likely an effect of steric hindrance. However, there was an increase in the relative α-2,3-sialylation in ovarian cancer samples as compared to healthy controls. Moreover, the relative α-2,3-linked sialylation increased with increasing antennarity and cancer stage, reaching its maximum in tetraantennary structures (50%).

Since the increase in total α-2,3/α-2,6-sialylation ratio was statistically significant for ovarian cancer, ROC curves were generated for sialylation ratios, the CA125 biomarker and for the binary logistic regression model of the combination of CA125 with the total sialylation ratio. The proposed model could improve the classification of both early- and late-stage ovarian cancer patients compared to CA125 alone. While CA125 alone showed a sensitivity of 84.4% and a specificity of 97%, in combination with the sialylation ratio, both sensitivity and specificity increased to 89.6% and 100%, respectively.

The advantage of such an approach lies in the utilization of a biomarker, which is already used all over the world in clinical laboratories, whose sensitivity and specificity could be improved by an additional measurement of glycosylation. Such a measurement could be proposed in unclear cases and/or cases below certain cut-off value of CA125. Since the results observed here were obtained from the whole serum N-glycome, it is unclear, which glycoproteins contributed to the changes, however the increased α-2,3-sialylation clearly correlated with ovarian cancer stage. A further sialylation study of specific proteins, such as acute-phase proteins, could shed light on the pathogenesis of ovarian cancer.

## Data Availability

The datasets generated for this study are available on request to the corresponding author.

## Ethics Statement

The ethical committee of the Charité Medical University, Berlin, Germany, approved the use of the samples (EA4/073/06 and EA1/285/09).

## Author Contributions

VB, EB, and JS contributed conception and design of the study. EB coordinated sample collection and database, TD performed the experiments and data analysis. All authors contributed to manuscript writing, revision. All authors read and approved the submitted version.

### Conflict of Interest Statement

The authors declare that the research was conducted in the absence of any commercial or financial relationships that could be construed as a potential conflict of interest.
