# Supplementary material for: Sialic Acid Linkage Analysis Refines the Diagnosis of Ovarian Cancer
Source: Front Oncol. 2019 Apr 11;9:261. doi: 10.3389/fonc.2019.00261 (PMC6499200; doi:10.3389/fonc.2019.00261)
Supplement: Supplementary file 1 [file Data_Sheet_1.docx]

Supplementary Table 1. Glycan trait calculation. H = hexose, N = N-acetylhexosamine, A = α-2,3-linked sialic acid, D = α-2,6-linked sialic acid, F = fucose.

| Glycan trait | Formula |
| --- | --- |
| High-mannose | N2H5 + N2H6 + N2H7 + N2H8 |
| Complex asialo | N3H4 + N4H3 + N4H5 + N5H4 + N5H5 + N5H6 |
| Complex asialo fucosylated | N4H3F1 + N4H4F1 + N4H5F1 + N5H4F1 + N5H5F1 + N5H5F2 + N5H6F1 |
| Hybrid | N3H5A1 + N3H6A1 + N3H5D1 + N3H6D1 |
| Complex monoantennary | N3H4A1 + N3H4D1 |
| Complex biantennary | N4H4A1 + N4H4D1 + N4H5A1 + N4H5D1 + N4H5A2 + N4H5D1A1 + N4H5D2 |
| Complex triantennary | N5H5D1 + N5H6A1 + N5H6D1 + N5H5D2 + N5H6D1A1 + N5H6D2 + N5H6A3 + N5H6D1A2 + N5H6D2A1 + N5H6D3 |
| Complex tetraantennary | N6H7D1A1 + N6H7D2 + N6H7D1A2 + N6H7D2A1 + N6H7D1A3 + N6H7D2A2 + N6H7D3A1 |
| Fucosylated complex biantennary | N4H5A1F1 + N4H5D1F1 + N4H5A2F1 + N4H5D1A1F1 + N4H5D2F1 |
| Fucosylated complex triantennary | N5H5D1F1 + N5H6D1F1 + N5H5D1A1F1 + N5H5D2F1 + N5H6D1A1F1 + N5H6D2F1 + N5H6D1A2F1 + N5H6D2A1F1 + N5H6D3F1 + N5H6D1A2F2 + N5H6D2A1F2 |
| Fucosylated complex tetraantennary | N6H7D1A1F1 + N6H7D1A2F1 + N6H7D2A1F1 + N6H7D1A3F1 + N6H7D2A2F1 + N6H7D3A1F1 + N6H7D1A3F2 + N6H7D2A2F2 |
| Biantennary α-2,3 | N4H4A1 + N4H5A1 + N4H5A2 + (1/2) * (N4H5D1A1) |
| Biantennary α-2,6 | N4H4D1 + N4H5D1 + (1/2) * (N4H5D1A1) |
| Triantennary α-2,3 | N5H6A1 + N5H6A3 + (1/2) * (N5H6D1A1) + (2/3) * (N5H6D1A2) + (1/3) * (N5H6D2A1) |
| Triantennary α-2,6 | N5H5D1 + N5H6D1 + N5H5D2 + N5H6D2 + N5H6D3 + (1/2) * (N5H6D1A1) + (1/3) * (N5H6D1A2) + (2/3) * (N5H6D2A1) |
| Tetraantennary α-2,3 | (1/2) * (N6H7D1A1 + N6H7D2A2) + (2/3) * (N6H7D1A2) + (1/3) * (N6H7D2A1) + (3/4) * (N6H7D1A3) + (1/4) * (N6H7D3A1) |
| Tetraantennary α-2,6 | (1/2) * (N6H7D1A1 + N6H7D2A2) + (1/3) * (N6H7D1A2) + (2/3) * (N6H7D2A1) + (1/4) * (N6H7D1A3) + (3/4) * (N6H7D3A1) + N6H7D2 |
| Fucosylated complex biantennary α-2,3 | N4H5A1F1 + N4H5A2F1 + (1/2) * (N4H5D1A1F1) |
| Fucosylated complex biantennary α-2,6 | N4H5D1F1 + N4H5D2F1 + (1/2) * (N4H5D1A1F1) |
| Fucosylated complex triantennary α-2,3 | (1/2) * (N5H5D1A1F1 + N5H6D1A1F1) + (2/3) * (N5H6D1A2F1) + (1/3) * (N5H6D2A1F1) |
| Fucosylated complex triantennary α-2,6 | N5H5D1F1 + N5H6D1F1 + N5H5D2F1 + N5H6D2F1 + N5H6D3F1 + (1/2) * (N5H5D1A1F1 + N5H6D1A1F1) + (1/3) * (N5H6D1A2F1) + (2/3) * (N5H6D2A1F1) |
| Fucosylated complex tetraantennary α-2,3 | (1/2) * (N6H7D1A1F1 + N6H7D2A2F1 + N6H7D2A2F2) + (2/3) * (N6H7D1A2F1) + (1/3) * (N6H7D2A1F1) + (3/4) * (N6H7D1A3F1 + N6H7D1A3F2) + (1/4) * (N6H7D3A1F1) |
| Fucosylated complex tetraantennary α-2,6 | (1/2) * (N6H7D1A1F1 + N6H7D2A2F1 + N6H7D2A2F2) + (1/3) * (N6H7D1A2F1) + (2/3) * (N6H7D2A1F1) + (1/4) * (N6H7D1A3F1 + N6H7D1A3F2) + (3/4) * (N6H7D3A1F1) |
| Fucosylated complex α-2,3 | Fucosylated complex biantennary α-2,3 + fucosylated complex triantennary α-2,3 + fucosylated complex tetraantennary α-2,3 |
| Fucosylated complex α-2,6 | Fucosylated complex biantennary α-2,6 + fucosylated complex triantennary α-2,6 + fucosylated complex tetraantennary α-2,6 |
| Afucosylated complex α-2,3 | Complex biantennary α-2,3 + complex triantennary α-2,3 + complex tetraantennary α-2,3 |
| Afucosylated complex α-2,6 | Complex biantennary α-2,6 + complex triantennary α-2,6 + complex tetraantennary α-2,6 |
| Complex α-2,3 | Fucosylated complex α-2,3 + afucosylated complex α-2,3 |
| Complex α-2,6 | Fucosylated complex α-2,6 + afucosylated complex α-2,6 |
| α-2,3/ α-2,6 ratio | sum all α-2,3 ratios / sum α-2,6 ratios |

Supplementary Table 2. Results of non-parametric T_JT_ of relative intensities of high-mannose structures and total high-mannosylation. Positive and negative Z values indicate increasing and decreasing trends, respectively. P values < 0.05 were considered statistically significant.

| **Glycan type** | **Glycan** | ***m/z*** | **T_JT_** | **Z value** | **p value** |
| --- | --- | --- | --- | --- | --- |
| **High-mannose** | N2H5 | 1257.4 | 637 | -6.764 | <0.0001 |
|  | N2H6 | 1419.5 | 1044 | -4.44 | <0.0001 |
|  | N2H7 | 1581.5 | 1885.5 | 0.367 | 0.714 |
|  | N2H8 | 1743.6 | 1634.5 | -1.068 | 0.286 |
|  | **Total** | **-** | **958.0** | **-4.931** | **< 0.0001** |

Supplementary Table 3. Results of non-parametric T_JT_ of relative intensities of hybrid N-glycan structures and their total relative intensity during ovarian cancer progression. Positive and negative Z values indicate increasing and decreasing trends, respectively. P values < 0.05 were considered statistically significant.

| **Glycan type** | **Glycan** | ***m/z*** | **T_JT_** | **Z value** | **p value** |
| --- | --- | --- | --- | --- | --- |
|  |  |  |  |  |  |
| **Hybrid-type** | N3H5A1 | 1750.6 | 783.0 | -5.930 | < 0.0001 |
|  | N3H5D1 | 1778.6 | 715.0 | -6.319 | < 0.0001 |
|  | N3H6A1 | 1912.7 | 2103.5 | 2.030 | 0.042 |
|  | N3H6D1 | 1940.7 | 1646.5 | -1.012 | 0.312 |
|  | Total | - | 767.0 | -6.022 | < 0.0001 |

Supplementary Table 4. Results of non-parametric T_JT_ of relative intensities of neutral complex-type N-glycans structures during ovarian cancer progression. Positive and negative Z values indicate increasing and decreasing trends, respectively. P values < 0.05 were considered statistically significant.

| **Glycan type** | **Glycan** | ***m/z*** | **T_JT_** | **Z value** | **p value** |
| --- | --- | --- | --- | --- | --- |
|  |  |  |  |  |  |
| **Neutral complex-type** | N3H4 | 1298.4 | 1478.5 | -2.086 | 0.037 |
|  | N4H3 | 1339.5 | 1891.5 | 0.411 | 0.681 |
|  | N4H5 | 1663.6 | 434.5 | -7.920 | < 0.0001 |
|  | N5H4 | 1704.6 | 2192.5 | 2.127 | 0.033 |
|  | N5H5 | 1866.7 | 2140.5 | 2.011 | 0.044 |
|  | N5H6 | 2028.7 | 2065.0 | 2.332 | 0.020 |
|  | Total | - | 1188.0 | -3.618 | 0.0003 |

Supplementary Table 5. Results of non-parametric T_JT_ of relative intensities of fucosylated complex-type neutral N-glycans structures during ovarian cancer progression. Positive and negative Z values indicate increasing and decreasing trends, respectively. P values < 0.05 were considered statistically significant.

| **Glycan type** | **Glycan** | ***m/z*** | **T_JT_** | **Z value** | **p value** |
| --- | --- | --- | --- | --- | --- |
|  |  |  |  |  |  |
| **Neutral complex fucosylated** | N4H3F1 | 1485.5 | 1919.0 | 0.557 | 0.578 |
|  | N4H4F1 | 1647.6 | 1310.0 | -2.921 | 0.003 |
|  | N4H5F1 | 1809.6 | 1005.0 | -4.662 | < 0.0001 |
|  | N5H4F1 | 1850.7 | 1126.0 | -3.972 | < 0.0001 |
|  | N5H5F1 | 2012.7 | 1604.0 | -1.246 | 0.213 |
|  | N5H5F2 | 2158.8 | 1857.0 | 0.203 | 0.839 |
|  | N5H6F1 | 2174.8 | 1966.5 | 1.232 | 0.218 |
|  | Total | - | 1579.0 | -1.385 | 0.166 |

Supplementary Table 6. Results of non-parametric T_JT_ of relative intensities of monoantennary sialylated N-glycan structures and their total relative intensity during ovarian cancer progression. Positive and negative Z values indicate increasing and decreasing trends, respectively. P values < 0.05 were considered statistically significant.

| **Glycan type** | **Glycan** | ***m/z*** | **T_JT_** | **Z value** | **p value** |
| --- | --- | --- | --- | --- | --- |
|  |  |  |  |  |  |
| **Complex sialylated monoantennary** | N3H4A1 | 1588.6 | 2865.0 | 6.224 | < 0.0001 |
|  | N3H4D1 | 1616.6 | 473.0 | -7.700 | < 0.0001 |
|  | Total | - | 1074.0 | -4.268 | < 0.0001 |

Supplementary Table 7. Results of non-parametric T_JT_ of relative intensities of biantennary sialylated N-glycan structures, sialic acid ratio and their total relative intensity during ovarian cancer progression. Positive and negative Z values indicate increasing and decreasing trends, respectively. P values < 0.05 were considered statistically significant.

| **Glycan type** | **Glycan** | ***m/z*** | **T_JT_** | **Z value** | **p value** |
| --- | --- | --- | --- | --- | --- |
|  |  |  |  |  |  |
| **Complex sialylated afucosylated biantennary** | N4H4A1 | 1791.6 | 2757.5 | 5.616 | < 0.0001 |
|  | N4H4D1 | 1819.7 | 1151.0 | -3.829 | 0.0001 |
|  | N4H5A1 | 1953.7 | 2034.5 | 1.216 | 0.224 |
|  | N4H5D1 | 1981.7 | 758.0 | -6.073 | < 0.0001 |
|  | N4H5A2 | 2243.8 | 1338.0 | -3.283 | 0.001 |
|  | N4H5D1A1 | 2271.8 | 1286.0 | -3.058 | 0.002 |
|  | N4H5D2 | 2299.9 | - | - | - |
|  | α-2,3/α-2,6 | - | 2947.0 | 6.427 | < 0.0001 |
|  | Total | - | 879.0 | -5.382 | < 0.0001 |
| **Complex sialylated fucosylated biantennary** | N4H5A1F1 | 2099.7 | 1029.0 | -4.531 | < 0.0001 |
|  | N4H5D1F1 | 2127.8 | 829.0 | -5.668 | < 0.0001 |
|  | N4H5A2F1 | 2389.9 | 1047.0 | -4.424 | < 0.0001 |
|  | N4H5D1A1F1 | 2417.9 | 2402.5 | 3.318 | 0.001 |
|  | N4H5D2F1 | 2445.9 | 784.0 | -5.924 | < 0.0001 |
|  | α-2,3/α-2,6 | - | 2391.0 | 3.252 | 0.0011 |
|  | Total | - | 750.0 | -6.119 | < 0.0001 |

Supplementary Table 8. Results of non-parametric T_JT_ of relative intensities of triantennary sialylated N-glycan structures, sialic acid ratio and their total relative intensity during ovarian cancer progression. Positive and negative Z values indicate increasing and decreasing trends, respectively. P values < 0.05 were considered statistically significant.

| **Glycan type** | **Glycan** | ***m/z*** | **T_JT_** | **Z value** | **p value** |
| --- | --- | --- | --- | --- | --- |
|  |  |  |  |  |  |
| **Complex sialylated Bisect/Triantennary** | N5H5D1 | 2184.8 | 1307.0 | -2.938 | 0.003 |
|  | N5H6A1 | 2318.8 | 1905.0 | 0.550 | 0.583 |
|  | N5H6D1 | 2346.9 | 1116.0 | -4.030 | < 0.0001 |
|  | N5H5D2 | 2502.9 | 1445.5 | -2.165 | 0.03 |
|  | N5H6D1A1 | 2637.0 | 1131.5 | -3.942 | < 0.0001 |
|  | N5H6D2 | 2665.0 | 876.0 | -5.399 | < 0.0001 |
|  | N5H6A3 | 2899.0 | 2247.5 | 3.618 | 0.0003 |
|  | N5H6D1A2 | 2927.1 | 1741.5 | -0.458 | 0.647 |
|  | N5H6D2A1 | 2955.1 | 1197.0 | -3.566 | 0.0004 |
|  | N5H6D3 | 2983.1 | 1819.0 | -0.014 | 0.989 |
|  | α-2,3/α-2,6 | - | 1562.0 | -1.482 | 0.138 |
|  | Total | - | 1229.0 | -3.383 | 0.001 |
| **Complex sialylated fucosylated Triantennary** | N5H5D1F1 | 2330.9 | 1266.0 | -3.172 | 0.002 |
|  | N5H6D1F1 | 2492.9 | 1591.0 | -1.624 | 0.104 |
|  | N5H5D1A1F1 | 2621.0 | 2052.5 | 1.645 | 0.100 |
|  | N5H5D2F1 | 2649.0 | 1194.0 | -3.583 | 0.0003 |
|  | N5H6D1A1F1 | 2783.0 | 2256.0 | 2.571 | 0.01 |
|  | N5H6D2F1 | 2811.1 | 1675.0 | -0.869 | 0.385 |
|  | N5H6D1A2F1 | 3073.1 | 2621.0 | 4.655 | < 0.0001 |
|  | N5H6D2A1F1 | 3101.2 | 2816.0 | 5.679 | < 0.0001 |
|  | N5H6D3F1 | 3129.2 | 1988.5 | 0.968 | 0.333 |
|  | N5H6D1A2F2 | 3219.2 | 2347.5 | 3.978 | < 0.0001 |
|  | N5H6D2A1F2 | 3247.2 | 2288.0 | 3.167 | 0.002 |
|  | α-2,3/α-2,6 | - | 2982.0 | 6.627 | < 0.0001 |
|  | Total | - | 2429.0 | 3.469 | 0.001 |

Supplementary Table 9. Results of non-parametric T_JT_ of relative intensities of tetraantennary sialylated N-glycan structures, sialic acid ratio and their total relative intensity during ovarian cancer progression. Positive and negative Z values indicate increasing and decreasing trends, respectively. P values < 0.05 were considered statistically significant.

| **Glycan type** | **Glycan** | ***m/z*** | **T_JT_** | **Z value** | **p value** |
| --- | --- | --- | --- | --- | --- |
|  |  |  |  |  |  |
| **Complex sialylated tetraantennary** | N6H7D1A1 | 3002.1 | 1880.5 | 0.345 | 0.73 |
|  | N6H7D2 | 3030.1 | 2259.0 | 3.024 | 0.002 |
|  | N6H7D1A2 | 3292.2 | 2112.0 | 1.804 | 0.071 |
|  | N6H7D2A1 | 3320.2 | 2021.0 | 1.228 | 0.219 |
|  | N6H7D1A3 | 3582.3 | 2267.0 | 2.839 | 0.005 |
|  | N6H7D2A2 | 3610.3 | 2149.5 | 1.901 | 0.057 |
|  | N6H7D3A1 | 3638.4 | 2320.5 | 3.308 | 0.001 |
|  | α-2,3/α-2,6 | - | 1726.5 | -0.546 | 0.585 |
|  | Total | - | 2248.5 | 2.446 | 0.014 |
| **Complex sialylated fucosylated Tetraantennary** | N6H7D1A1F1 | 3148.2 | 2346.0 | 3.865 | 0.0001 |
|  | N6H7D1A2F1 | 3438.3 | 2306.5 | 3.834 | 0.0001 |
|  | N6H7D2A1F1 | 3466.3 | 2242.0 | 3.502 | 0.0005 |
|  | N6H7D1A3F1 | 3728.4 | 2428.0 | 4.366 | < 0.0001 |
|  | N6H7D2A2F1 | 3756.4 | 2746.0 | 5.565 | < 0.0001 |
|  | N6H7D3A1F1 | 3784.4 | 2390.5 | 4.364 | < 0.0001 |
|  | N6H7D1A3F2 | 3874.4 | 2224.5 | 3.423 | 0.001 |
|  | N6H7D2A2F2 | 3902.5 | 2318.5 | 3.929 | < 0.0001 |
|  | α-2,3/α-2,6 | - | 490.5 | 0.663 | 0.507 |
|  | Total | - | 2864.0 | 6.184 | < 0.0001 |

Supplementary Table 10. Results of non-parametric T_JT_ of sialic acid ratio and total relative intensity of sialylated N-glycan structures during ovarian cancer progression. Positive and negative Z values indicate increasing and decreasing trends, respectively. P values < 0.05 were considered statistically significant.

| **Glycan type** | **Fucosylation** | **Glycan** | **T_JT_** | **Z value** | **p value** |
| --- | --- | --- | --- | --- | --- |
|  |  |  |  |  |  |
| **Complex sialylated** | **Afucosylated** | α-2,3/α-2,6 | 2705.0 | 5.045 | < 0.0001 |
|  |  | Total | 948.0 | -4.988 | < 0.0001 |
|  | **Fucosylated** | α-2,3/α-2,6 | 2934.0 | 6.353 | < 0.0001 |
|  |  | Total | 1815.0 | -0.037 | 0.970 |
|  | **Total** | α-2,3/α-2,6 | 3057.0 | 7.055 | < 0.0001 |
|  |  |  |  |  |  |


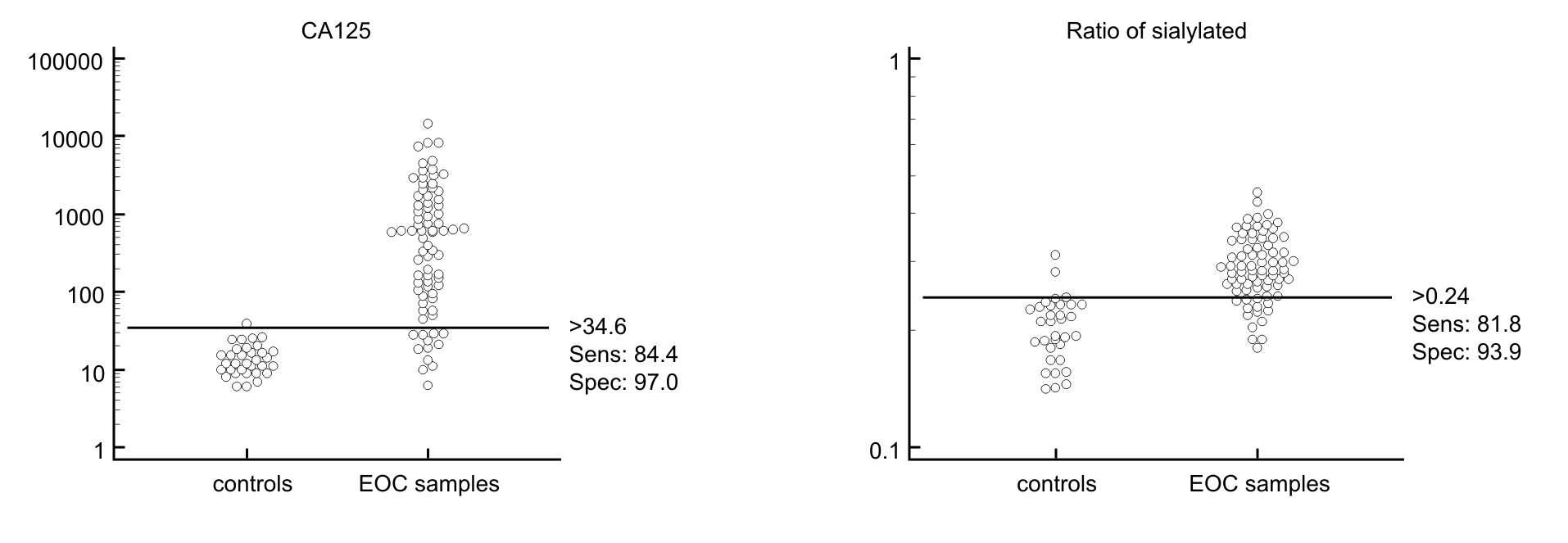


Supplementary Figure 1. Dot plot of logarithmically transformed values, lines indicate sensitivity and specificity at the calculated cut-off values.


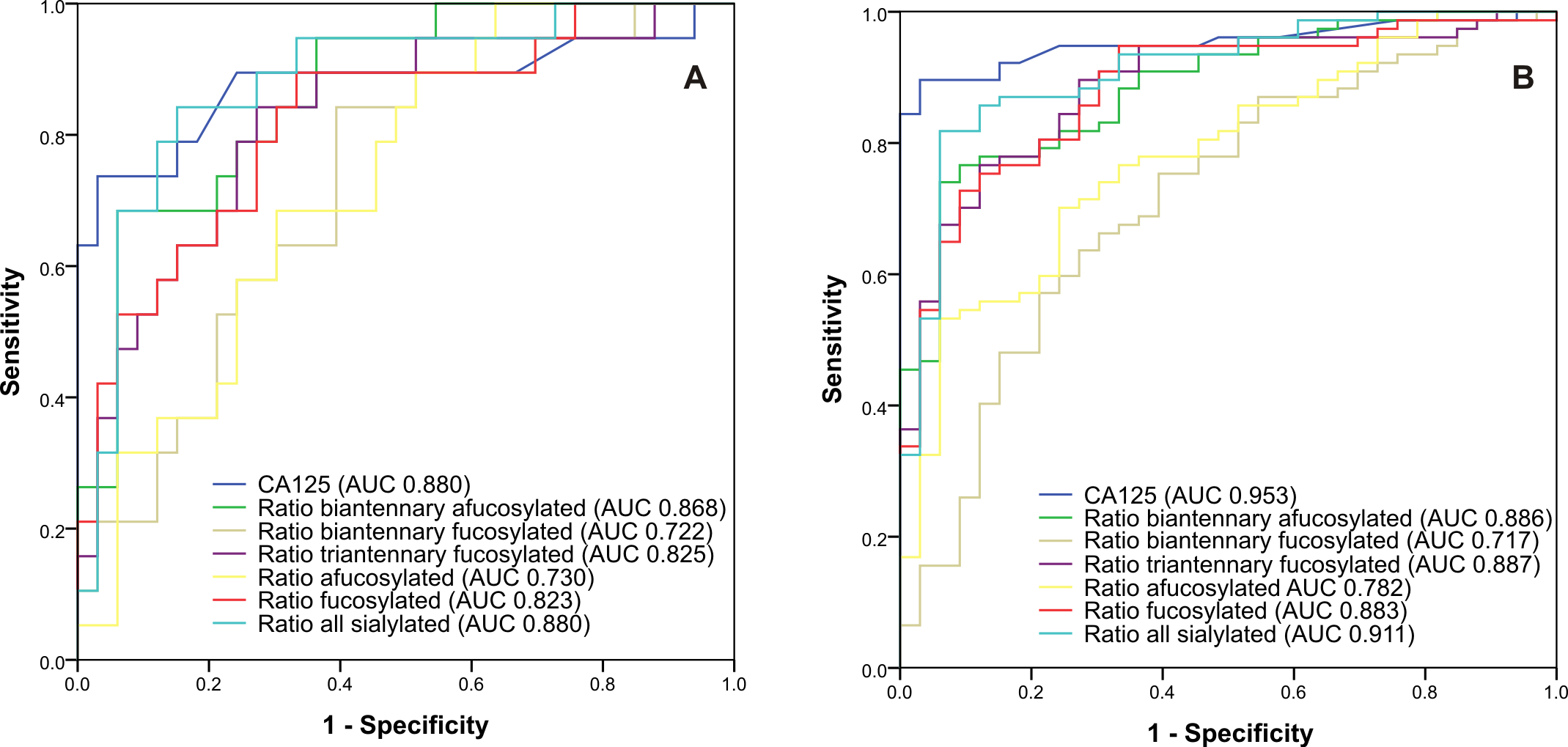


Supplementary Figure 2. ROC curves of various sialylation ratios and CA125 taken separately generated for A) 33 healthy controls and 19 early-stage ovarian cancer patients B) 33 healthy controls and 77 ovarian cancer patients (early- and late-stage patients).
